# A Novel CRB2 Mutation Associated With FSGS and ESRD in an Adult Patient

**DOI:** 10.1155/crin/8140153

**Published:** 2026-05-20

**Authors:** Michele Marchini, Sonila Mocka, Matteo Trezzi

**Affiliations:** ^1^ Department of Nephrology, Azienda Sociosanitaria Ligure 5, La Spezia, 19121, Italy

**Keywords:** chronic kidney disease, CRB2 protein, focal segmental glomerulosclerosis, mutation, podocytes, whole exome sequencing

## Abstract

**Background:**

Chronic kidney disease (CKD) is a major global health concern, with a substantial proportion of cases that remain of undetermined cause. Mutations in genes affecting podocyte structure and function, are increasingly recognized as causes of focal segmental glomerulosclerosis (FSGS), a common but highly nonspecific histological pattern of kidney injury, that ultimately lead to CKD.

**Case Presentation:**

We report the case of a 55‐year‐old male who presented with hypertension and end‐stage renal disease (ESRD) of unknown etiology. He had a progressive decline in kidney function and proteinuria beginning in young adulthood. A kidney biopsy showed a pattern of FSGS. A comprehensive workup did not identify autoimmune or inflammatory causes. Whole‐exome sequencing detected a previously undescribed heterozygous CRB2 mutation (c.1037G > T, p.Cys346Phe), predicted to be deleterious.

**Discussion:**

Animal models of CRB2 deprivation in podocytes showed progression toward FSGS. In humans, CRB2 mutations have previously been linked to severe early‐onset nephrotic syndrome, typically in homozygous or compound heterozygous states. This is the first report of an adult‐onset CRB2‐associated FSGS in a heterozygous state, suggesting a milder disease course with a progressive kidney decline. As with other genetic forms of FSGS, we hypothesize that heterozygous CRB2 mutations may permit near‐normal renal function for years until cumulative stressors trigger podocyte injury and CKD progression.

**Conclusion:**

This case expands the clinical spectrum of CRB2‐related kidney disease and highlights the importance of genetic testing in adults with unexplained CKD. Identifying genetic forms of CKD may refine diagnostic and therapeutic approaches in nephrology.

## 1. Introduction

Chronic kidney disease (CKD) is a major global health burden, with the prevalence and associated morbidity projected to rise substantially in the coming decades [1]. CKD encompasses a heterogeneous group of conditions that impair kidney structure and function and may ultimately progress to end‐stage renal disease (ESRD). Despite advances in diagnostic strategies, a precise etiological diagnosis often remains elusive: A substantial proportion of patients initiating kidney replacement therapy (KRT) lacks a clearly identified cause of CKD. This diagnostic uncertainty negatively affects patient management and long‐term outcomes. Over the past decade, genetic causes of CKD have gained increasing recognition. International initiatives, including those led by KDIGO, have advocated for the integration of genomic testing into routine nephrology practice. Recent studies indicate that a significant proportion of individuals requiring KRT without a specific prior diagnosis actually harbor undetected monogenic kidney disorders [2]. These findings highlight the expanding role of genomic diagnostics not only in pediatric nephrology but also in adults, where monogenic diseases are now estimated to account for a meaningful percentage of CKD cases. Among genetic kidney diseases, focal segmental glomerulosclerosis (FSGS) represents the most frequently observed histologic pattern of injury. FSGS is characterized by segmental sclerosis, hyalinosis, and adhesions affecting portions of the glomerular tuft, ultimately leading to podocyte loss. Importantly, FSGS is not a single disease entity but a histologic pattern that may arise from diverse etiologies, including autoimmune, genetic, viral, drug‐induced, and maladaptive causes, and is also a major cause of nephrotic syndrome in both children and adults. Although numerous genes involved in the podocyte structure and function have already been linked to monogenic forms of FSGS, the number of implicated variants continues to expand, underscoring the importance of genetic evaluation in patients with an FSGS pattern of injury [3]. Globally, the prevalence of CKD is estimated to be approximately 850 million cases, with approximately 4 million individuals needing KRT for kidney failure. By 2050, CKD is projected to become the fifth leading underlying cause of death worldwide [4]. In Europe, up to 27% of patients on KRT have no defined etiology for their renal disease, and similar figures are reported in the United States, where 18% of KRT patients remain without a known cause [5].

### 1.1. Genetics of FSGS and the Role of Crumbs2 (CRB2) in the Kidney

Over the past two decades, advances in genomic nephrology have identified mutations in numerous genes as causes of monogenic FSGS and steroid‐resistant nephrotic syndrome (SRNS) [6]. These genes predominantly encode proteins essential for podocyte structure, cytoskeletal regulation, and slit diaphragm integrity. Disruption of these finely coordinated mechanisms compromises the glomerular filtration barrier, ultimately leading to podocyte injury, proteinuria, and progressive kidney dysfunction. More than 50 nuclear genes have been implicated in monogenic forms of FSGS [7]. They include genes encoding structural components of the slit diaphragm—such as nephrin (NPHS1) and podocin (NPHS2) [8, 9], proteins‐regulating cell junctions and polarity (e.g., COL4A3, COL4A4, COL4A5, and LAMA5), and proteins encoded by genes involved in cytoskeletal dynamics (TRPC6, PLCE1, ACTN4, and MYH9) [10–12] and cytoskeletal regulation (ARHGAP24, ARHGDIA, and ANLN) [13–15]. Defects in genes required for Coenzyme Q10 biosynthesis, a conserved mitochondrial metabolic pathway, also lead to genetic forms of FSGS characterized by early and severe renal impairment [16]. Mutations in transcription factors (WT1, SMARCAL1, and LMX1B) [17–19] and nuclear pore complex proteins (NUP93, NUP107, NUP205, and XPO5) [20, 21] have also been associated with familial or sporadic FSGS. In addition, APOL1 represents a major susceptibility gene, where carriage of the G1 or G2 risk alleles confers substantially increased risk of FSGS in individuals of African ancestry when combined with environmental “second hits” [22, 23]. Mitochondrial DNA mutations have similarly been linked to podocyte loss and progressive CKD [24]. Historically, genetic testing for FSGS primarily targeted pediatric patients presenting with early‐onset nephrotic syndrome and rapid progression to ESRD. However, accumulating evidence demonstrates that monogenic FSGS may also present in adulthood, with broad variability in age of onset, proteinuria severity, and penetrance. Among adults of Western European descent, compound heterozygous variants in NPHS2—often involving the R229Q allele—represent one of the most frequent genotypes associated with adult‐onset FSGS [25–27]. Other genes linked to adult disease include INF2, the leading cause of autosomal‐dominant FSGS in familial cases [28–30], as well as, CD2AP, which are implicated in both sporadic and inherited disease [31, 32]. Because a considerable proportion of adults with CKD still lack a specific etiologic diagnosis, it is likely that unrecognized monogenic FSGS remains underdiagnosed. Improved genetic characterization of adult‐onset FSGS may ultimately refine diagnostic pathways and guide individualized therapeutic strategies.

CRB2 belongs to the evolutionarily conserved Crumbs family of polarity proteins, which regulate apical–basal organization and cell–cell junctions [33, 34]. In mammals, three isoforms exist—CRB1, CRB2, and CRB3—each with tissue‐specific expression patterns. CRB2 is highly expressed in tissues with polarized epithelial cells, such as the brain [35], retina [36], and podocyte [37]. In the kidney, it plays a pivotal role in the development and function of the glomerular filtration barrier, particularly in podocytes, the highly specialized cells responsible for the fine architecture of the slit diaphragm. It is now established that CRB2 stabilizes intercellular junctions and regulates cytoskeletal dynamics, ensuring the structural cohesion of podocytes to the protein of the slit diaphragm. CRB2’s function in cellular organization and organogenesis underscores its importance in both development and homeostasis. Knockdown studies in zebrafish demonstrated that loss of CRB2 homologs leads to disruption of podocyte foot processes and the absence of slit‐diaphragm structures [38]. Approximately a decade ago, biallelic pathogenic variants in the human gene CRB2 were identified as a cause of childhood‐onset SRNS and FSGS, with clinical features resembling congenital nephrotic syndrome caused by NPHS1 mutations, but often accompanied by extrarenal manifestations such as ventriculomegaly and elevated α‐fetoprotein [39]. Subsequent studies demonstrated that CRB2 is a core slit‐diaphragm protein interacting with nephrin, and that this interaction is essential to maintain filtration barrier integrity. In murine models, loss of CRB2 results in severe proteinuria, podocyte effacement, and FSGS, highlighting its critical role in glomerular filtration comparable to that of nephrin [40, 41].

## 2. Case Report

Clinical Presentation: We report the case of a 55‐year‐old man who presented with hypertension and ESRD of unknown etiology. His clinical history revealed the onset of progressive CKD and subnephrotic proteinuria in his early thirties. He had no known family history of kidney disease or diabetes and no history of ocular, auditory, or cardiac disorders. There was no history of consanguinity in the patient’s family, and no personal or family history of diabetes. Complement levels (C3 and C4) were within the normal range. Body mass index was within normal limits, and the patient denied current or previous smoking history. Screening for autoimmune and inflammatory conditions yielded negative results. Nongenetic secondary causes of FSGS, such viral infections, and conditions associated with maladaptive glomerular hyperfiltration such as low birth weight, were investigated and excluded based on clinical history and laboratory evaluation. Early renal and urinary tract imaging studies were unremarkable. No extrarenal manifestations were reported.

**FIGURE 1:**
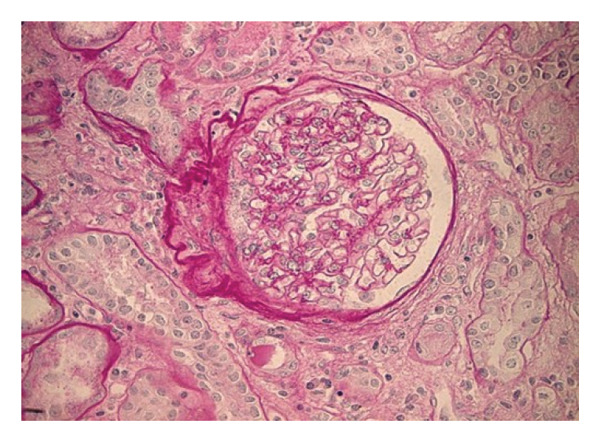
Hematoxylin and eosin stain showing focal and segmental sclerosis with partial obliteration of capillary loops.

**FIGURE 2:**
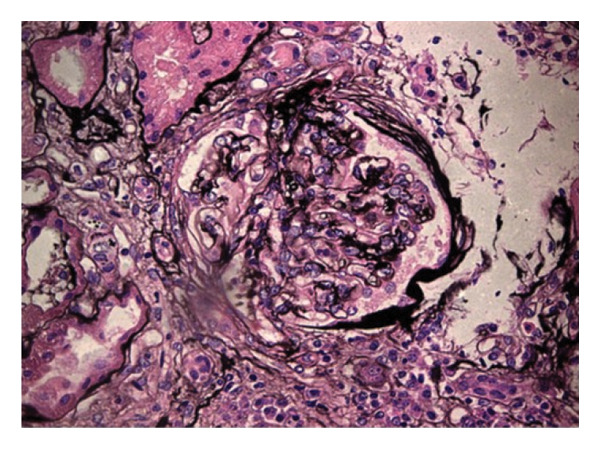
Methenamine silver stain highlighting capillary loop obliteration and increased extracellular matrix deposition.

**FIGURE 3:**
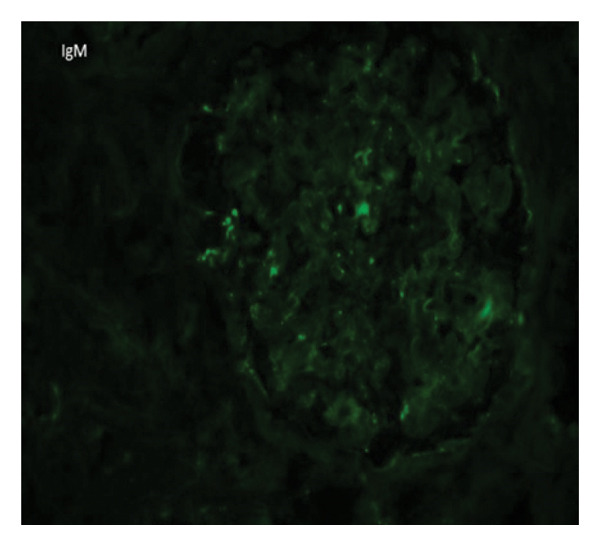
Immunofluorescence for IgM showing mild, nonspecific staining restricted to sclerotic glomerular segments.

**FIGURE 4:**
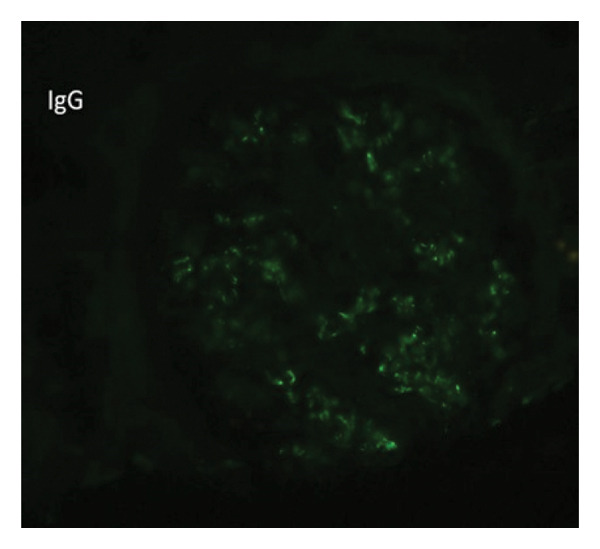
Immunofluorescence for IgG showing mild, focal, nonspecific staining in sclerotic glomerular segments.

**FIGURE 5:**
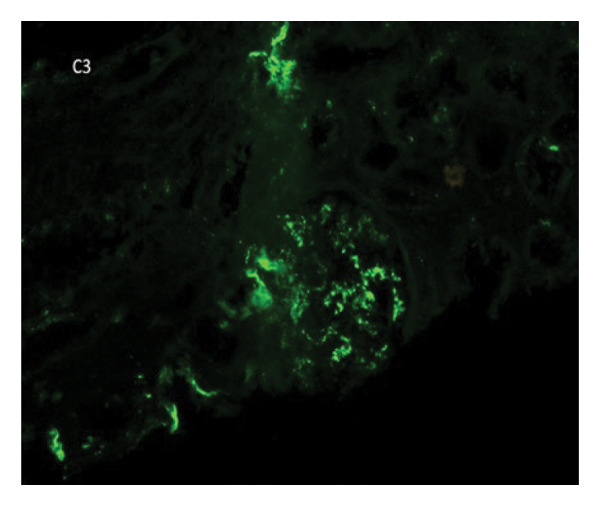
Immunofluorescence for C3 demonstrating mild, nonspecific deposition in sclerotic glomeruli and tubules.

Kidney Biopsy Findings: Kidney biopsy demonstrated FSGS with segmental sclerosis involving portions of the glomerular tuft and adhesions to Bowman’s capsule. Hematoxylin and eosin and methenamine silver staining highlighted segmental capillary loop obliteration and increased mesangial matrix deposition (Figures 1 and 2). Immunofluorescence (IF) revealed mild, focal, nonspecific IgG, IgM, and C3 staining limited to sclerotic segments (Figures 3, 4, and 5). On this basis, the patient was diagnosed with CKD due to not‐otherwise‐specified FSGS (NOS‐FSGS).

**FIGURE 6:**
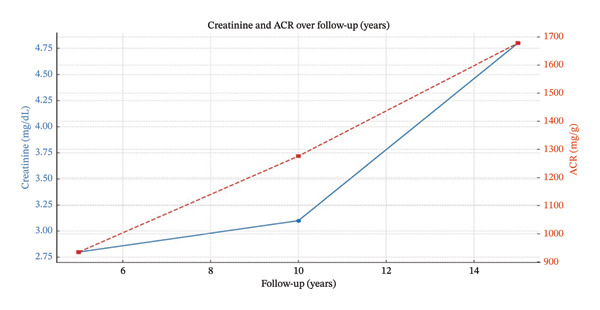
Progression of patient’s chronic kidney disease over the years of follow‐up and the trend of proteinuria measured as albumin–creatinine ratio (ACR) in mg/gr.

Follow‐up and Clinical Course: The patient was started on renin–angiotensin–aldosterone system inhibition (RAASi) at the maximum tolerated dose. Given the absence of features suggestive of an immune‐mediated form of FSGS, together with subnephrotic proteinuria and a slowly progressive course, immunosuppressive therapy was not initiated after careful evaluation of the potential risks and benefits. Management consisted primarily of supportive care and RAASi with the aim of controlling hypertension and possibly slowing disease progression. Over the following years, serum creatinine demonstrated a modest but progressive increase, while proteinuria remained persistently below the nephrotic range. Serial ultrasound studies showed a gradual, symmetric reduction in kidney size. CKD progressed until approximately 50 years of age, when he reached ESRD with a creatinine level of 4.5 mg/dL and an estimated glomerular filtration rate (eGFR) of 14 mL/min/1.73 m^2^. Longitudinal trends in kidney function and proteinuria are summarized in Figure 6.

Genetic Testing: Given the absence of identifiable secondary causes and the biopsy findings, whole‐exome sequencing (WES) was performed. WES identified a previously undescribed heterozygous variant in CRB2: c.1037G > T p.(Cys346Phe). This variant has not been reported in the literature or major genomic databases. In silico prediction tools supported a deleterious effect (PolyPhen‐2: 1.00; SIFT: 0.00; MutationTaster: 1.00; CADD‐PHRED: 28.4). Conservation metrics also indicated strong evolutionary constraint at this position (phyloP‐Vertebrate: 5.22; phyloP‐Primate: 0.55; PhastCons: 1.00). The genetic findings were reviewed by a clinical geneticist. Sanger confirmation was not performed, as the analysis was conducted in a research‐based context and the variant was reported based on the NGS data. According to the current ACMG criteria, the variant should be classified as a variant of uncertain significance (VUS); however, given the computational predictions and the clinical context, it may represent a likely deleterious candidate variant contributing to the patient’s phenotype. WES did not identify pathogenic variants in other genes known to be associated with FSGS or hereditary podocytopathies, including a panel of 159 genes linked to monogenic forms of FSGS and SRNS. These findings are summarized in Table 1. Based on the clinical history, kidney biopsy findings, and the absence of alternative etiologies, the CRB2 variant was considered the most plausible contributor to the patient’s CKD.

**TABLE 1:** In silico predictions and conservation scores of the CRB2 variant.

Gene locus	Nucleotide alteration	Amino acid alteration	SIFT	PolyPhen‐2	PhyloP	CADD‐PHRED	Mutation taster	NHLBI exome variant server
CRB2 9q33.3	c.1037G > T	p.(Cys346Phe)	0.0/0.00	1.0/1.00	0.55/0.65	28.4	1.00/1.00	Not present

## 3. Discussion

CRB2 is a polarity protein essential for maintaining podocyte architecture and for stabilizing slit‐diaphragm components through interactions with nephrin and the actin cytoskeleton. Disruption of CRB2 impairs podocyte organization and filtration barrier integrity, leading to proteinuria and progressive glomerular damage. Our case describes a 55‐year‐old man carrying a heterozygous CRB2 variant who developed adult‐onset FSGS with a slowly progressive course. The identified variant c.1037G > T, p.(Cys346Phe) substitutes a conserved cysteine residue with phenylalanine. Cysteine residues within extracellular domains commonly participate in the disulfide bond formation, which is critical for proper protein folding and structural stability. Disruption of these residues may therefore impair CRB2 structural integrity and potentially contribute to podocyte dysfunction. This finding is notable because all previously reported CRB2‐associated glomerular diseases have involved biallelic pathogenic variants and have presented exclusively in childhood, typically with severe phenotypes ranging from congenital nephrotic syndrome to SRNS. Unlike homozygous or compound heterozygous CRB2 mutations, heterozygous mutations, which likely reflect residual CRB2 activity, may permit near‐normal renal function for an extended period before cumulative stressor, such as aging, comorbidities, or environmental factors, exacerbate podocyte injury and precipitate progressive renal function decline toward ESRD. Cystic or microcystic kidney changes have been reported in several CRB2‐associated nephropathies, particularly in syndromic or early‐onset forms characterized by biallelic pathogenic variants. In contrast, cystic changes in our patient were not detected. Nevertheless, the absence of renal cysts in our case may reflect the heterozygous genetic background and the later onset of disease; therefore, the absence of cystic changes should not necessarily exclude the possibility of CRB2‐related nephropathy. The presence of a heterozygous mutation in CRB2 may provide a plausible explanation for the clinical features of our case, such as the late onset, the non‐nephrotic proteinuria, absence of cysts, and the slow progression to ESRD. Overall, our report possibly expands the clinical spectrum of CRB2‐related disease and raises the possibility that heterozygous CRB2 variants may contribute to milder, late‐onset podocytopathies. We speculate that as genetic testing becomes more widely accessible, the identification of genetic forms of FSGS due to heterozygous CRB2 mutations in adults will be more commonly described.

## Funding

This study was supported by the Department of Science and Technology of Jilin Province, 20250102243JC

## Consent

Written informed consent was obtained from the patient for publication of this case report.

## Conflicts of Interest

The authors declare no conflicts of interest.

## Data Availability

The data that support the findings of this study are available on request from the corresponding author. The data are not publicly available due to privacy or ethical restrictions.
